# Comparison of laser in situ ketatomileusis and photorefractive keratectomy for myopia using a mixed-effects model

**DOI:** 10.1371/journal.pone.0174810

**Published:** 2017-03-31

**Authors:** Yosai Mori, Kazunori Miyata, Takashi Ono, Yusuke Yagi, Kazutaka Kamiya, Shiro Amano

**Affiliations:** 1 Miyata Eye Hospital, Miyakonojo, Japan; 2 Department of Ophthalmology, University of Kitasato School of Medicine, Kanagawa, Japan; 3 Inouye Eye Hospital, Tokyo, Japan; Bascom Palmer Eye Institute, UNITED STATES

## Abstract

**Purpose:**

To compare the results of laser in situ keratomileusis (LASIK) and photorefractive keratectomy (PRK) for myopia using a mixed-effects model.

**Methods:**

This comparative retrospective study was conducted in 1,127 eyes of 579 patients after LASIK and 270 eyes of 144 patients after PRK who had two or more postoperative follow-ups after 3 months. Uncorrected visual acuity (UCVA), best spectacle-corrected visual acuity (BSCVA), manifest refractive spherical equivalent (MRSE), percentage of eyes within ± 0.5 diopters (D) and ± 1.0 D of targeted refraction, and central corneal thickness were compared between PRK and LASIK groups using a mixed-effects model.

**Results:**

Compared with the LASIK group, UCVA in the PRK group was significantly worse in the initial year but was significantly better after 4 years. The average BSCVA was not significantly different between the LASIK and PRK groups after 4 years. The average gain of BSCVA in the PRK group was significantly larger than that of the LASIK group after 2 years. MRSE in the LASIK and PRK groups showed a gradual myopic shift until 6 years after surgery. After 6 years, MRSE in the PRK group remained stable whereas MRSE in the LASIK group continued a myopic shift. The percentages of eyes within ± 0.5 D or ± 1.0 D in the LASIK group were significantly higher than those in the PRK group at 3 months but were significantly lower than those in the PRK group at 10 years.

**Conclusions:**

PRK for myopia shows better efficacy than LASIK for myopia after 4 years.

## Introduction

Laser in situ keratomileusis (LASIK) and photorefractive keratectomy (PRK) have been the most frequently performed refractive surgeries, and the results of long-term follow-ups as long as 10 years have been reported.[1–11] Some of these previous studies involved only patients who completed the long-term follow-up. However, a selection bias likely occurs when patients with missing data or incomplete follow-ups are excluded. On the other hand, when patients with missing data are included, bias because of missing data is inevitable. Recently, the mixed-effects model has been used for analyses of longitudinal clinical data to reduce the bias involving selected or missing data. We therefore conducted a study to compare the results of LASIK and PRK using this model.

## Materials and methods

The study protocol adhered to the tenets of the Declaration of Helsinki. The institutional review board of Miyata Eye Hospital approved this retrospective study and waived the requirement for informed consent. The study involved consecutive patients who underwent LASIK or PRK at Miyata Eye Hospital to correct myopia. LASIK was performed from March 2000 to March 2015, and PRK was performed from May 1998 to July 2013. Inclusion criteria included primary LASIK or PRK, a refractive target being emmetropia, and two or more postoperative follow-ups after 3 months. Exclusion criteria included patients < 19 years of age. When patients underwent retreatment or other ocular surgeries after LASIK or PRK, the data up to these surgeries were included in the study. When the patients underwent LASIK or PRK on both eyes, the data from both eyes were included.

### Surgical technique

In the LASIK procedure, a microkeratome (MK-2000; Nidek, Aichi, Japan) with a 160-μm head was used to create an 8.5-mm corneal flap with a nasal hinge. Laser ablation was performed with a VISX Star S2 or Star S4 excimer laser (AMO, Santa Ana, CA, USA). In all procedures, a 6.0-mm optical zone and conventional ablation were used. After surgery, 0.1% fluorometholone and 0.5% levofloxacin eye drops were prescribed four times daily, starting the day after surgery, for 2 weeks, three times daily for 2 weeks, and then twice daily for 1 month.

In the PRK procedure, the central 6.0-mm diameter epithelium was ablated with an excimer laser. Laser ablation was performed with a VISX Star S or Star S2 excimer laser (AMO). After PRK laser treatment, a bandage soft contact lens was placed on the cornea. Eye drops containing 0.1% betamethasone sodium phosphate (Shionogi Pharmaceutical, Osaka, Japan) and 0.5% levofloxacin (Santen Pharmaceutical, Osaka, Japan) were prescribed four times daily starting the day after surgery for 2 weeks, After 2 weeks, the 0.1% betamethasone sodium phosphate eye drops were terminated, and 0.1% fluorometholone eye drops (Santen Pharmaceutical) were prescribed four times daily for 3 months, three times daily for 2 months, and then twice daily for 2 months.

### Measurements

Examinations included uncorrected visual acuity (UCVA), best spectacle-corrected visual acuity (BSCVA), manifest refraction, intraocular pressure, and central corneal thickness (CCT) using an ultrasound pachymeter (SP-2000; Tomey, Nagoya, Japan). The data from these examinations before and at 3, 6, 12, and 24 months after surgery, and once every 2 years thereafter, were recorded from the medical charts.

### Statistical analysis

Comparison measurements of UCVA, BSCVA, manifest refractive spherical equivalent (MRSE), and change of CCT between the LASIK and PRK groups at each time point were performed using a mixed-effects model with time, treatment, and time by treatment interaction as fixed effects and with patients and eyes within patients as random effects.[12,13]

The measured time series percentages of eyes whose MRSE was within ± 0.5 diopters (D) and ± 1.0 D were estimated using generalized estimating equation models with the Logit Link Function with time, treatment, and time by treatment interaction as explanatory variables and with exchangeable correlation structure within eyes/patients.

## Results

A retrospective comparison was conducted on 1,127 eyes of 579 patients after conventional LASIK and 270 eyes of 144 patients after PRK. Preoperative characteristics of the patients are shown in Table 1. The patient groups were comparable in age, manifest refraction, and corneal refractive power. Preoperative BSCVA was significantly better in the LASIK group than in the PRK group. During the 10 postoperative years, 69 eyes (6.1%) of 40 patients after LASIK and 9 eyes (3.3%) of 5 patients after PRK underwent retreatment.

**Table 1:** Demographic data.

	LASIK	PRK	p-value
No. of patients (no. of eyes)	579 (1,127)	144 (270)	
Age (years)	33.7±9.3	33.3±10.8	0.60
Sex (male/female)	236/343	75/69	0.0147
Ablation depth (μm)	63.8±25.5	67.1±27.0	0.058
Pupil diameter (mm)	6.5±0.8	6.3±0.9	0.0008
logMAR UCVA	1.36±0.29	1.38±0.26	0.25
logMAR BSCVA	-0.19±0.09	-0.09±0.11	<0.0001
Sphere (D)	-5.82±2.64	-5.49±2.65	0.068
Cylinder (D)	-0.94±0.94	-0.96±1.02	0.71
Spherical equivalent (D)	-6.29±2.71	-5.97±2.64	0.086
Mean keratometry (D)	43.92±1.45	44.02±1.69	0.31
Keratometric cylinder (D)	1.41±0.84	1.41±0.99	0.99

LASIK = laser in situ keratomileusis; PRK = photorefractive keratectomy; no. = number; logMAR = logarithm of the minimum angle of resolution; UCVA = uncorrected visual acuity; BSCVA = best spectacle-corrected visual acuity; D = diopters.

### Visual acuity

**Fig 1:**
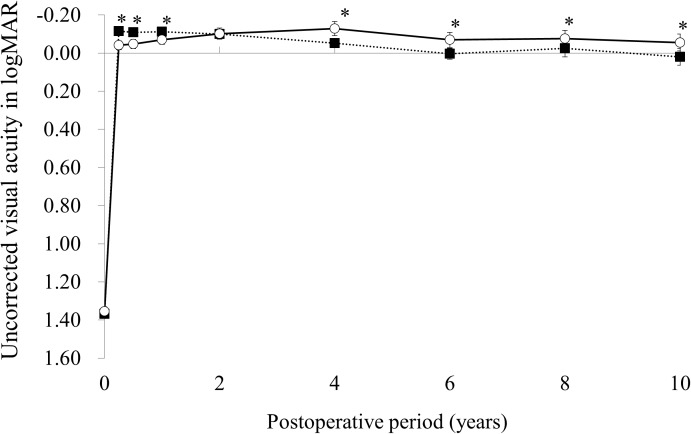
Time courses of the average and 95% confidence intervals of uncorrected visual acuity in the laser in situ keratomileusis (closed square) and photorefractive keratectomy (open square) groups. * p< .05.

The time courses of the average and 95% confidence intervals of UCVA in the LASIK and PRK groups are shown in Fig 1. UCVA in the LASIK group gradually decreased whereas UCVA in the PRK group gradually increased during the initial 4 years, decreased from 4 to 6 years, and remained stable after 6 years. The average UCVA was significantly better in the LASIK group than in the PRK group at 3 months (p < 0.0001), 6 months (p < 0.0001), and 1 year (p < 0.0001). In contrast, the average UCVA was significantly better in the PRK group than in the LASIK group at 4 years (p = 0.0002), 6 years (p = 0.0093), 8 years (p = 0.0169), and 10 years (p = 0.0011).

**Fig 2:**
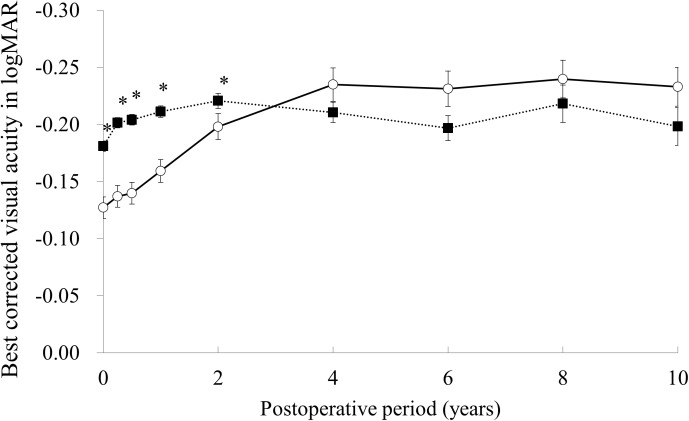
Time courses of the average and 95% confidence intervals of best spectacle-corrected visual acuity in the laser in situ keratomileusis (closed square) and photorefractive keratectomy (open square) groups. * p< .05.

The time courses of the average and 95% confidence intervals of BSCVA in the LASIK and PRK groups are shown in Fig 2. The average BSCVA in the LASIK group was stable during the 10-year follow-up whereas the average BSCVA in the PRK group gradually increased during the initial 4 years after surgery. The average BSCVA in the LASIK group was significantly better than that of the PRK group at 3 months, 6 months, 1 year, and 2 years (all, p < 0.0001). After 4 years, there was no significant difference in the average BSCVA between the LASIK and PRK groups.

**Fig 3:**
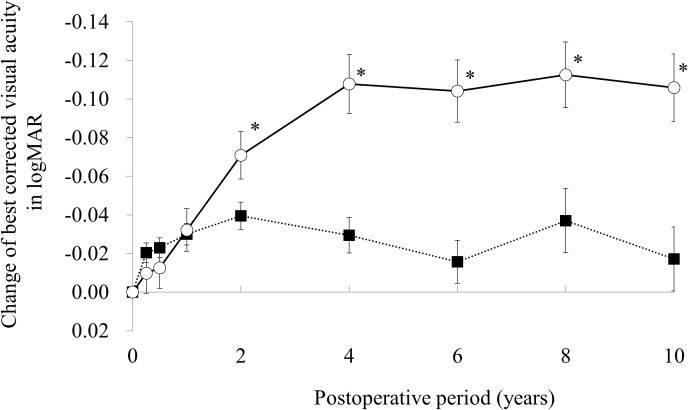
Time courses of the average and 95% confidence intervals of change of best spectacle-corrected visual acuity in the laser in situ keratomileusis (closed square) and photorefractive keratectomy (open square) groups. * p< .05.

Time courses of the average and 95% confidence intervals of change of BSCVA from preoperation in the LASIK and PRK groups are shown in Fig 3. The average gain of BSCVA in the PRK group was significantly larger than that of the LASIK group after 2 years.

### Manifest refraction

**Fig 4:**
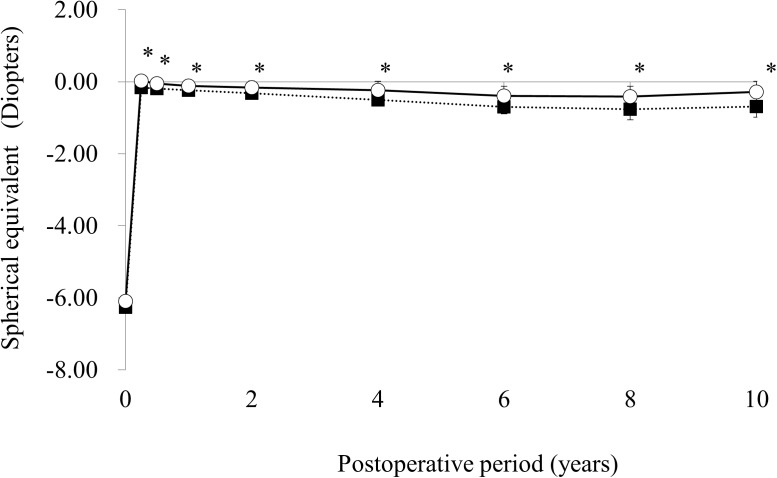
Time courses of the average and 95% confidence intervals of manifest refractive spherical equivalent in the laser in situ keratomileusis (closed square) and photorefractive keratectomy (open square) groups. * p< .05.

The time courses of the average MRSE and 95% confidence intervals are shown in Fig 4. The regression and regression rate in the LASIK and PRK groups are shown in Table 2. MRSE in the LASIK and PRK groups showed gradual myopic shifts until 6 years after surgery. After 6 years, MRSE in the PRK group remained stable whereas MRSE in the LASIK group continued a myopic shift. The average MRSE in the LASIK group was significantly more myopic than that in the PRK group at all time points (all, p < 0.0001).

**Table 2:** Mean values of regression and regression rates after LASIK and PRK.

	LASIK	PRK	p-value
Regression (diopters)			
3M to 10Y	-0.656	-0.409	0.0006
1Y to 10Y	-0.593	-0.277	<0.0001
2Y to 10Y	-0.525	-0.191	<0.0001
4Y to 10Y	-0.380	-0.119	0.0011
6Y to 10Y	-0.263	+0.031	0.0004
8Y to 10Y	-0.124	+0.070	0.032
Regression per year (dioters/year)			
3M to 10Y	-0.067	-0.042	0.0006
1Y to 10Y	-0.066	-0.031	<0.0001
3M to 1Y	-0.084	-0.175	0.059
1Y to 2Y	-0.068	-0.086	0.685
2Y to 4Y	-0.072	-0.036	0.203
4Y to 6Y	-0.059	-0.075	0.625
6Y to 8Y	-0.069	-0.020	0.225
8Y to 10Y	-0.062	+0.035	0.032

LASIK = laser in situ keratomileusis; PRK = photorefractive keratectomy; Y = years; M = months.

The estimated percentages of eyes within ± 0.5 D and ± 1.0 D are shown in Table 3. In the LASIK group, the percentages of eyes within ± 0.5 D or ±1.0 D decreased over time whereas those in the PRK group remained almost stable. At 3 months, the percentages of eyes within ± 0.5 D or ±1.0 D in the LASIK group were significantly higher than those in the PRK group. In contrast, at 10 years, the percentages of eyes within ± 0.5 D or ± 1.0 D in the LASIK group were significantly lower than those in the PRK group.

**Table 3:** Estimated percentages of eyes within ± 0.5 and ± 1.0 diopters.

	Within ±0.5 diopters			Within ±1.0 diopters		
	LASIK	PRK	p-values	LASIK	PRK	p-values
3 M	80.5	74.0	0.018	95.1	90.1	0.0020
6M	81.2	77.4	0.161	94.9	89.9	0.0035
1Y	79.7	80.1	0.876	96.3	92.7	0.0233
2Y	75.7	72.9	0.461	92.8	92.8	0.993
4Y	61.5	79.2	0.0016	88.5	95.4	0.069
6Y	58.8	66.3	0.263	83.9	87.0	0.522
8Y	48.3	60.8	0.120	76.9	80.7	0.555
10Y	47.0	75.5	0.0006	73.4	89.6	0.0087

LASIK = laser in situ keratomileusis; PRK = photorefractive keratectomy; Y = years; M = months.

### Retreatment

After LASIK, 59 (5.2%) of 1,127 eyes underwent retreatment whereas 9 (3.3%) of 270 eyes underwent retreatment after PRK. In the LASIK group, 27 of 59 eyes underwent retreatment between 6 months and 1 year, and 32 eyes between 1 and 9 years. In the PRK group, 9 eyes underwent retreatment after 1 year.

### Corneal thickness

**Fig 5:**
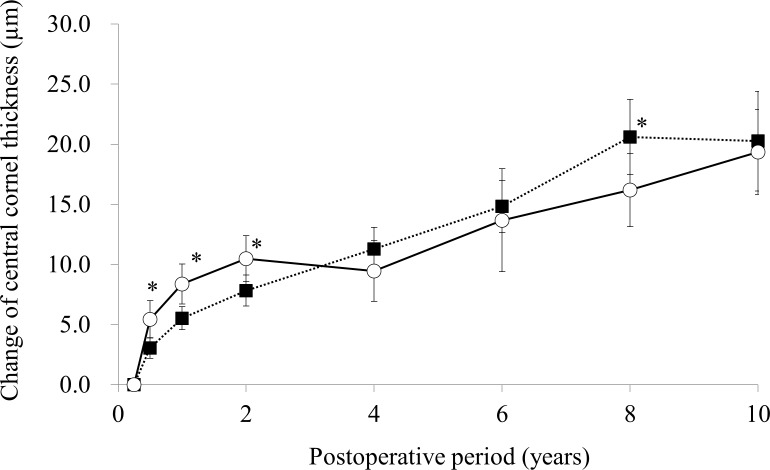
Time courses of the average and 95% confidence intervals of change of central corneal thickness from 3 months in the laser in situ keratomileusis (closed square) and photorefractive keratectomy (open square) groups. * p< .05.

The time courses of the average and 95% confidence intervals of CCT change from 3 months in the LASIK and PRK groups are shown in Fig 5. The average change of CCT in the LASIK group continuously increased during the follow-up, but that in the PRK group increased for the initial 2 years and stayed stable until 4 years, then increased again from 4 years. Compared with the LASIK group, the average change of CCT in the PRK group was significantly greater at 6 months (p = 0.0084), 1 year (p = 0.0036), and 2 years (p = 0.023), and was significantly smaller at 8 years (p = 0.048)

## Discussion

After LASIK and PRK, patients with positive outcomes may think that it is unnecessary to attend their follow-up appointments. Conversely, patients with complications may want a different follow-up care, and changes in the clinic that they visit. Thus, clinical studies of LASIK and PRK tend to have many patients with incomplete follow-ups and/or missing data, and these selection processes probably affect the results of clinical studies of LASIK and PRK. The mixed-effects model provides a good method of analyses of studies with uncomplete follow-ups and/or missing data. The results of the mixed-effects model in the present study indicated that MRSE in the LASIK and PRK groups showed a gradual myopic shift until 6 years after surgery, and MRSE in the PRK group remained stable after 6 years, whereas MRSE in the LASIK group continued a myopic shift. These results in the PRK group differ from our previous study that included only patients with complete 4-year follow-ups.[14] The results showed that MRSE in the PRK group remained stable during 4 years of follow-up and MRSE in the LASIK group showed a gradual myopic shift. The differences in the analyses of missing data and the statistical methods used likely resulted in differences between our previous study and the current study.

As shown in Table 2, the regression rates of MRSE from 3 months to 10 years in the LASIK and PRK groups were −0.064 diopters/year and −0.045 diopters/year, respectively. Alió et al calculated the same indices from 3 months to 10 years in their studies and showed that the regression rates of MRSE after LASIK were −0.10 D/year in 97 patients with a mean preoperative MRSE of −7.27 D and −0.18 D/year in 196 patients with a mean preoperative MRSE of −13.95 D.[4,5] Their results suggested that the regression rate has a positive correlation with the corrected refractive power. Considering that the mean preoperative MRSE of patients in the LASIK group in the present study was −6.29 D, the regression rate of −0.064 D/year is compatible with the results of Alió et al. In other studies of PRK by Alió et al, the regression rates of MRSE from 3 months to 10 years were −0.01 D/year in 225 patients with a mean preoperative MRSE of −3.81 D and −0.07 D/year in 267 patients with a mean preoperative MRSE of −8.87 D.[7,8] Again, the regression rate of −0.045 D/year in the PRK group in the current study is compatible with studies by Alió et al when considering that the mean preoperative MRSE of patients in the PRK group was −5.97 D.

The retreatment rate in the present study was higher for LASIK than for PRK. In the LASIK group, 27 (2.4%) of 59 eyes underwent retreatment between 6 months and 1 year, and 32 (2.8%) eyes between 1 year and 9 years, suggesting the cause of retreatment was undercorrection in approximately half of the patients, with myopic regression in the other patients. In the PRK group, 9 (3.3%) eyes underwent retreatment after 1 year, suggesting the cause of retreatment was myopic regression in all cases. Thus, the continuous myopic regression caused the necessity for retreatment almost equally in the LASIK and PRK groups. However, the retreatments owing to undercorrection were only observed in the LASIK group, suggesting the excimer laser nomograms used for this study were a better fit for the PRK.

When the regression rates at different postoperative periods were calculated (Table 2), the regression rates in the LASIK group were similar throughout the postoperative period. However, the regression rates in the PRK group were relatively large during the first 2 years and decreased after 6 years. These results suggested that LASIK and PRK have different wound-healing patterns in a long-term postoperative period, possibly owing to the presence/absence of the corneal flap. Wound healing under the corneal flap may be prolonged after LASIK. On the other hand, wound healing after PRK was accelerated during the initial 2 years, decelerated during 2–4 years, accelerated during 4–6 years, and got stable after 6 years. Although the causes for the repetition of acceleration and deceleration of wound healing after PRK was unclear, the wound healing process seemed to get stable earlier after PRK than after LASIK.

The average change of CCT in the LASIK group continuously increased until 8 years. This continuous increase is consistent with the continuous myopic regression in the LASIK group, and the continuous changes in both CCT and MRSE are likely owing to prolonged wound-healing processes in the cornea after LASIK. Erie et al reported that keratocyte density around the flap interface is less than that in other areas of the corneal stroma 5 years after LASIK, suggesting that the extracellular matrix is produced at the flap interface and results in a lower keratocyte density.[15] This finding suggests that newly produced extracellular matrix at the flap interface increases the CCT and results in a steeper anterior corneal surface, with a resultant myopic shift.

In the LASIK group, the changes in MRSE and CCT corresponded with each other. However, the changes in MRSE and CCT in the PRK group showed different patterns. In particular, MRSE remained stable after 6 years, whereas CTT increased from 4 to 10 years. Because CCT in the PRK group remained stable between 2 and 4 years, the wound-healing process stabilized at 2 years after surgery. Thus, the increase of CCT after 4 years in the PRK group is surprising. Moreover, the discrepancy between increasing CCT and stable MRSE between 6 and 10 years is difficult to explain. Investigations of long-term wound-healing processes after PRK in clinical cases and animal models are therefore necessary to elucidate the mechanisms for CCT increases 6 years after PRK.

In this study, the average UCVA was significantly better in the LASIK group than in the PRK group during the initial first year. This superiority in UCVA of LASIK over PRK in short-term efficacy agrees with previous reports.[16–22] However, the average UCVA in the LASIK group decreased whereas the average UCVA in the PRK group increased from 3 months to 4 years, and the average UCVA in the PRK was better than that of the LASIK group after 4 years. Additionally, the percentages of eyes within ± 0.5 D or ± 1.0 D in the LASIK group were significantly higher than those in the PRK group at 3 months but were significantly lower than those in the PRK group at 10 years. Thus, the superiority of LASIK over PRK in UCVA, at least in our patients, was not retained 4 years after surgery. This continuous decline of UCVA in the LASIK group may result mainly from continuous myopic regression.

The average BSCVA in the LASIK group was significantly better than that of the PRK group until 2 years after surgery. This result is consistent with previous reports by us and others.[14, 16–22] Since the baseline BSCVA was significantly better in the LASIK group than in the PRK group, the direct comparison of postoperative BSCVA in the two groups is not appropriate. Hence, the change of BSCVA from preoperation in the two groups was compared and the result showed that the average gain of BSCVA in the PRK group was significantly larger than that of the LASIK group after 2 years. This result suggest that PRK has better safety to LASIK.

Alió et al compared the long-term outcomes of LASIK and PRK for myopia between −6 and −10 D, and found that LASIK had slightly better efficacy, predictability, and a lower rate of retreatment after 10 years.[1] Their results disagree with our present study showing that PRK had better mean UCVA than LASIK after 10 years. In the study by Alio et al,[1] 34 eyes after LASIK and 34 eyes after PRK with complete follow-up were selected from 4,800 eyes that underwent LASIK or PRK. However, in the present report, data from all eyes with at least two follow-ups after 3 months were included, and the mixed-effects model was used for data analyses. These differences in the patient selection and data analyses likely resulted in different conclusions. Moreover, the differences in race and postoperative medication may be other causes for the varying results between the two studies.

A limitation of this study was its retrospective nature. Although a prospective randomized controlled study with complete long-term follow-ups is ideal, the ratio of dropout cases is likely to increase as the follow-up period increases. Thus, the bias of selection and missing data might also affect a prospective study with a long-term follow-up. In the present study, a mixed-effects model was used to analyze the results of a retrospective study with incomplete follow-up and missing data.

In conclusion, both the LASIK and PRK groups showed gradual myopic shifts until 6 years after the surgery, which continued until 10 years in the LASIK group and stabilized in the PRK group. UCVA in the PRK group was worse than that in the LASIK group at 1 year, but was better than that in the LASIK group after 4 years. Hence, PRK for myopia shows better efficacy than LASIK for myopia after 4 years. Patients undergoing myopic correction should be informed of the long-term follow-up results of LASIK versus PRK.
